# Simultaneous Preoperative Prediction of Locally Advanced Breast Cancer, DCIS Component, and Multifocality Using Structured Mammographic Features and Gradient-Boosting Machine Learning

**DOI:** 10.3390/diagnostics16172744

**Published:** 2026-08-27

**Authors:** Sorour Raeiskarimi, Mahdi Saeedi-Moghadam, Fariba Zarei, Banafsheh Zeinali-Rafsanjani

**Affiliations:** 1 Student Research Committee, School of Medicine, Shiraz University of Medical Sciences, Shiraz 71936-35899, Iran; soroorraiskarimi79@gmail.com; 2 Medical Imaging Research Center, Shiraz University of Medical Sciences, Shiraz 71936-35899, Iran; m_saeedimoghadam@yahoo.com; 3 Department of Radiology, Shiraz University of Medical Sciences, Shiraz 71936-35899, Iran

**Keywords:** breast cancer, locally advanced breast cancer, mammography, machine learning, gradient boosting, SHAP, decision curve analysis, preoperative risk stratification

## Abstract

**Background/Objectives:** Accurate preoperative detection of locally advanced breast cancer is essential for neoadjuvant therapy planning. We developed and validated gradient-boosting models using structured BI-RADS mammographic features to simultaneously predict locally advanced breast cancer (LABC), DCIS component, and multifocality in a multi-center cohort. **Methods:** This retrospective study enrolled 2295 patients from three university-affiliated hospitals; features were coded according to BI-RADS. CatBoost and logistic regression models were built using stratified 60/20/20 splits, with performance assessed via bootstrap resampling, nested cross-validation, and sensitivity analyses. AUROC, AUPRC, Brier score, and calibration metrics assessed discrimination and clinical utility; a leakage audit and SHAP analysis supported interpretation. **Results:** CatBoost achieved an AUROC of 0.906 (95% CI: 0.876–0.932) for LABC. Because several top predictors overlap with the anatomical criteria defining this outcome, we repeated the analysis excluding them; the reduced model retained a mean AUROC of 0.739, indicating genuine predictive signal beyond the staging overlap. Net benefit was positive across all relevant thresholds, with calibration error of 0.053. DCIS prediction was highly accurate (AUROC 0.979; nested AUROC 0.9707), with no evidence of leakage. Multifocality prediction was more modest (AUROC 0.810), reflecting known limits of two-dimensional mammography. Sensitivity analyses confirmed stable performance across splits, training sizes, and class-weighting schemes. **Conclusions:** Structured mammographic features combined with gradient-boosting support clinically meaningful, though partly overlapping, risk stratification for LABC; once accounted for, the model still retains independent value. The DCIS model performed very well; multifocality prediction remains more limited, and external validation is needed before clinical use.

## 1. Introduction

Breast cancer is the most frequently diagnosed cancer among women worldwide and the second leading cause of cancer-related death for women. For women with locally advanced breast cancer, neoadjuvant chemotherapy (NAC) is the standard of care. NAC is used to facilitate tumor downstaging and provide in vivo assessment of treatment sensitivity [1]. Unfortunately, the response to NAC can vary significantly depending on tumor biology. Patients who develop progressive disease while receiving neoadjuvant therapy are at risk of becoming ineligible for curative surgery and have a higher likelihood of irreversible adverse clinical outcomes, such as tumor metastasis. Therefore, accurate preoperative risk stratification is vital for guiding treatment choice and identifying which patients may benefit from alternative or more intensive approaches [2].

Recent improvements in prognostic modeling for breast cancer have incorporated diverse data types. A combined system using breast MRI, whole slide images, and patient clinical information achieved an AUC of 0.909 for predicting pathological complete response in treatment-naïve locally advanced breast cancer patients, significantly outperforming models based on a single modality [3].

In a similar study, mathematical models based on contrast-enhanced and diffusion-weighted MRI predicted tumor response to neoadjuvant therapy in I-SPY 2 patients [4]. Additionally, nomograms incorporating dynamic changes in serum tumor markers improved response prediction, achieving an AUC of 0.739 in a training cohort [5]. Although these results demonstrate substantial potential, such methodologies require sophisticated imaging technologies and extensive computational resources that may limit widespread clinical implementation.

Beyond staging for neoadjuvant therapy, several tumor characteristics directly shape surgical decisions and what patients experience after treatment. One such characteristic is the presence of ductal carcinoma in situ (DCIS), which accounts for roughly one in four newly diagnosed breast cancers in screening-detected populations [6]. DCIS affects whether a surgeon can achieve clear margins during breast-conserving surgery and whether re-excision may be required [6,7]. Mammography is excellent at spotting calcified DCIS—most patients with high-grade DCIS show suspicious microcalcifications on their mammograms [8]. Knowledge about DCIS before surgery helps surgeons plan how much tissue to remove, aiming for clear margins and fewer repeat surgeries.

Another critical feature is multifocality—when cancer appears as two or more separate spots within the same breast. This matters because patients with multifocal disease have traditionally been considered candidates for mastectomy; however, breast-conserving surgery may be feasible in selected multifocal patients with adequate breast volume, while unifocal disease more consistently qualifies for breast-conserving surgery [9]. Furthermore, multifocal disease carries a significantly higher risk of sentinel lymph node metastases—up to 45% in multifocal cases compared to 28% in unifocal disease—representing nearly a 1.6-fold increase [10]. Consequently, preoperative assessment of multifocal disease is essential for planning the right operation.

Mammography stands apart from advanced imaging techniques because it is the most accessible and widely used breast cancer imaging tool worldwide [11]. The Breast Imaging Reporting and Data System (BI-RADS) gives radiologists a common language to describe what they see—mass shape and edges, calcification patterns, asymmetries, architectural distortion, and other features. These structured descriptions hold a wealth of clinically useful information that we might harness for preoperative risk prediction. Recent studies have already shown that machine learning models using mammographic features can predict DCIS recurrence (AUC 0.918) [6] and lymph node metastasis [10] with good accuracy. To date, no one has systematically asked whether these same mammographic features can predict locally advanced disease, DCIS component, and multifocality together—three outcomes that directly guide whether a patient receives neoadjuvant therapy and what kind of surgery they undergo.

Consequently, we developed and validated machine learning models using structured mammographic characteristics to preoperatively predict locally advanced breast cancer (primary outcome), DCIS component, and multifocality (secondary outcomes) in a multi-center cohort of 2295 patients from three university-affiliated hospitals. The novelty of the present study lies not in the individual methods employed, which include established tools such as CatBoost, logistic regression, SHAP, and decision curve analysis, but in their systematic application to the simultaneous, multi-outcome preoperative risk stratification of three clinically important breast cancer characteristics using routinely available, structured BI-RADS mammographic data, employing rigorous methodologies including 2000 bootstrap iterations, nested cross-validation for unbiased performance estimates, comprehensive sensitivity analyses, and decision curve analysis to evaluate clinical utility. A systematic feature leakage audit was performed to ensure no target-derived features were inadvertently included. Additionally, to improve model interpretability and identify the most informative features to guide neoadjuvant therapy decisions and surgical planning, we conducted SHAP (SHapley Additive exPlanations) analyses on the models developed from structured mammographic features combined with gradient-boosting machine learning techniques. As part of this rigor, we subsequently conducted dedicated incorporation-bias sensitivity analyses for both the locally advanced disease and DCIS component models—explicitly quantifying, rather than assuming away, the extent to which top-ranked predictors overlapped with each outcome’s definition (Section 2.14, Section 2.15, Section 3.3.5 and Section 3.4.5)—a check that is, to our knowledge, uncommon in comparable mammographic machine-learning studies.

## 2. Material and Methods

### 2.1. Study Design and Sample

The purpose of this study was to analyze and describe previously collected breast imaging data from three university-affiliated facilities over 2018 to 2025. This included all female patients aged 17 years and older (one patient aged 17 was included following individual institutional ethics board approval), who had at least one positive finding on digital mammography (mass, asymmetry, calcification, and/or architectural distortion).

**Figure 1:**
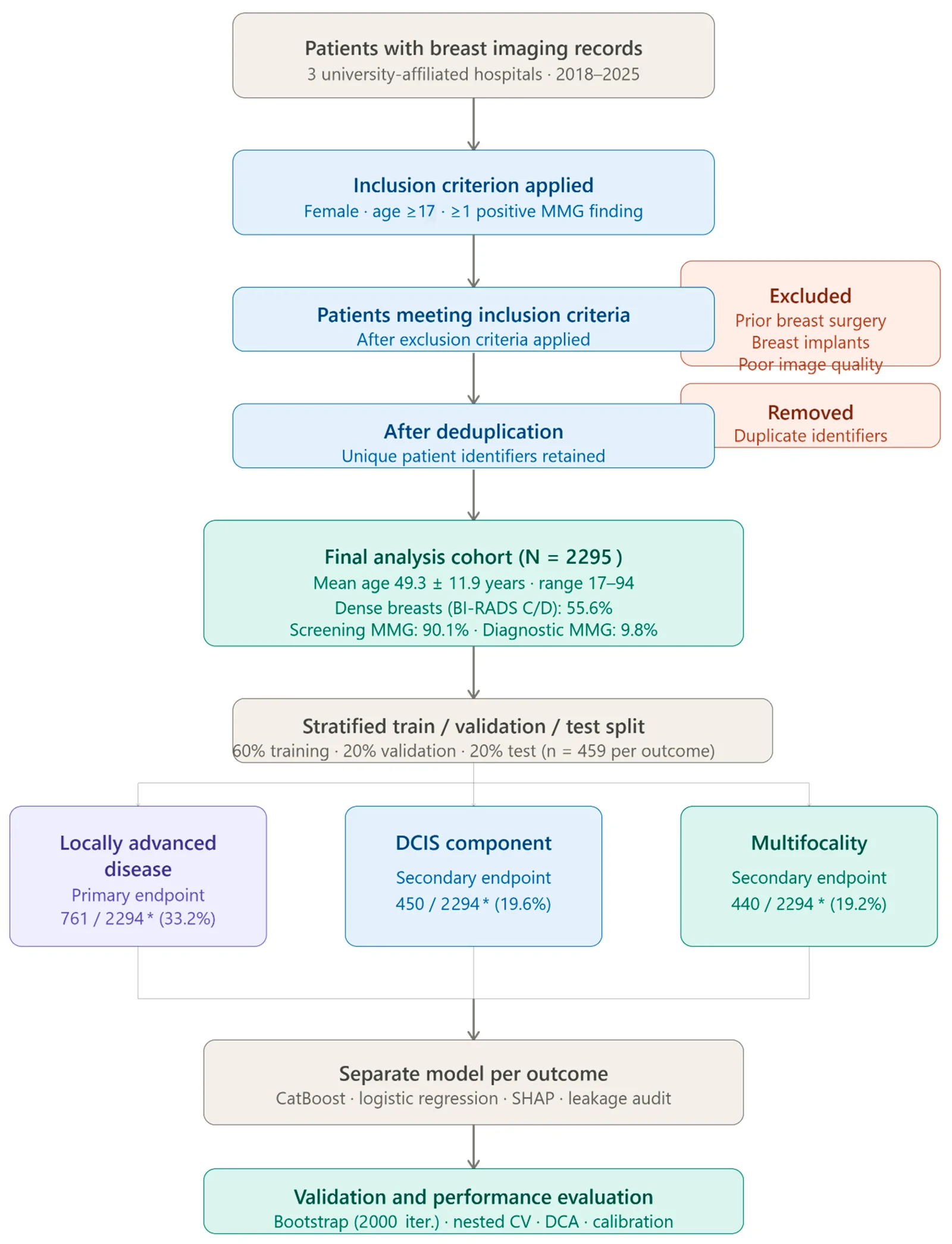
Patient selection flowchart. The final analysis cohort comprised 2295 patients from three university-affiliated hospitals (2018–2025). Outcome data were available for 2294 patients; one patient had no evaluable outcome recorded. Outcomes are non-mutually exclusive—a single patient may contribute to more than one endpoint simultaneously. * One patient had missing age but was retained in all outcome analyses (age-related calculations use *n* = 2294).

Exclusion criteria included having prior breast surgery, having breast implants, or having poor quality images that would not permit proper standard reporting. Mammographic screening at the participating institutions is not restricted by a strict lower age threshold; women referred by a clinician, presenting with breast symptoms, or with a positive family history were eligible for mammography regardless of age. This eligibility pattern reflects opportunistic, real-world clinical referral—driven by presenting symptoms, clinician referral, or a positive family history—rather than adherence to a formal, fixed-age population screening program; it was specified prospectively as an inclusion criterion and applied consistently across all three participating sites. Of the 2294 patients with available age data, 481 (21.0%) were under 40 years of age (including 194 under 35), of whom 457 (95.0%) underwent screening mammography, and 24 (5.0%) underwent diagnostic mammography. One patient aged 17 underwent screening mammography and had a positive mammographic finding; this patient was retained in the cohort. After removing duplicate identifiers, there were 2295 patients that made it to the final dataset. Of the 2295 patients in the final cohort, age data were available for 2294; the one patient with missing age was retained in all outcome analyses. Outcome prevalence is therefore reported with a denominator of 2294, as this patient contributed to the outcome dataset but not to age-related calculations (Figure 1), which shows the patient selection flowchart. The study received ethical approval from the Research Ethics Committee of Shiraz University of Medical Sciences (IR.SUMS.MED.REC.1403.423). Because we looked at retrospective data, we received a waiver of informed consent from each of the participating institutions.

Generative AI statement: During the preparation of this manuscript, Generative AI tools, including Claude 3.5 Sonnet and ChatGPT-4o, were used to assist with the writing and structuring of the manuscript. NotebookLM was used to assist in creating the Graphical Abstract. All AI-assisted content was reviewed and edited by the authors, who take full responsibility for the final content of the manuscript.

### 2.2. Data Extraction and Mammographic Features

Demographic information (patient age at mammography) and mammographic data were obtained from the PACS of each participating institution. One breast radiologist with considerable experience read all mammograms and reported findings using the BI-RADS lexicon, which provided consistency across participating sites [12,13].

The breast composition (almost entirely fatty; scattered fibroglandular densities; heterogeneous fibroglandular densities; or extremely dense) and breast mass characteristics were documented as subcategories according to how they fit into one of the following categories: morphology, margin, density, and size (less than 10 mm; 10–20 mm; 21–50 mm; greater than 50 mm) at each of the three participating facilities.

Calcification features were assessed by morphology (fine pleomorphic, coarse heterogeneous, or other) and distribution (segmental, regional, grouped, linear, or diffuse), as well as by accompanying asymmetry (asymmetry, focal asymmetry, global asymmetry, or developing asymmetry). The type and location of architectural distortion were also documented; additionally, other associated features such as skin thickening, skin retraction, nipple retraction, and axillary lymphadenopathy would also be recorded. Physical exam findings (e.g., presence of palpable masses) would also be recorded. Each mass that had similar characteristics would have been documented with separate identifiers, for example: (mass 1, mass 2, mass 3). The patients’ receptor status (ER, PR, HER2) was not included in the dataset and was therefore excluded from analysis. This represents a notable limitation, as receptor status is among the most important determinants of neoadjuvant chemotherapy eligibility and response, and its absence limits the model’s power for treatment decision-making.

### 2.3. Outcome Definitions

Three endpoints were examined in this study. The primary endpoint was designated as locally advanced disease because it directly pertained to neoadjuvant therapy decision-making and subsequent disease staging. The evaluation of DCIS component and multifocality were designated as secondary endpoints.

**Locally Advanced Disease (Primary Endpoint):** This was defined as breast cancer meeting one or more of the following criteria: tumor size greater than 50 mm (T3); direct extension to the chest wall or skin (T4a–c); inflammatory carcinoma (T4d); or clinically positive axillary lymph nodes (N1–N3). Axillary lymph node status was assessed by clinical examination; additional imaging with ultrasound or MRI was used where clinically indicated and available. This was recorded as a binary variable indicating the presence of a locally advanced area. Identifying locally advanced status preoperatively helps determine the patient’s eligibility for neoadjuvant systemic therapy and the surgical approach.

**DCIS Component (Secondary Endpoint):** This was defined as the presence of pure ductal carcinoma in situ (DCIS) with or without associated invasive cancer. This was recorded as a binary variable indicating DCIS component observed. The presence of DCIS is valuable to the operating room as it contributes to decisions made when surgical margins are evaluated and may predict the need for re-excision.

**Multifocality (Secondary Endpoint):** This was defined as the presence of two or more foci of invasive cancer or DCIS, confirmed by histopathological examination after core needle biopsy or surgical excision, and classified as multifocal (within the same breast quadrant) or multicentric (in different quadrants). This was recorded as a binary variable indicating the presence of multifocal lesions and influences surgical management (mastectomy versus breast-conserving surgery). All outcomes were determined from histopathological examination of biopsy or surgical specimens, with data extracted from pathology reports.

### 2.4. Feature Preprocessing and Leakage Prevention

Data were handled according to strict procedures to prevent invalid data from being used for clinical feature selection. Feature sets were determined for each outcome type based on clinical and methodological considerations.

The following were general exclusion rules for all models: (1) Deduplication of data was done through Patient ID. (2) All other outcome variables were eliminated from outcome datasets in order to prevent leakage of information. (3) Features derived from the pathology report were not retained since outcomes were based on pathology. (4) Inflammatory extent was not retained in the database as the definition of inflammatory disease overlaps with the definition of locally advanced disease.

The following features were retained for the primary endpoint (locally advanced disease): mass number and other mass features, as these contribute to disease burden and disease-stage determination. Mass number was recorded as a raw count (0–3 masses per patient) and entered as an ordinal categorical variable in model training; it was not used as a defining criterion for locally advanced classification, which was based exclusively on T- and N-stage criteria as described above. Among locally advanced patients (*n* = 761), 71.0% had a single mass, 9.9% had two masses, and 7.5% had three masses; the remaining 11.7% had no discrete mass and presented instead with calcifications, asymmetry, or architectural distortion. The same features were retained for the DCIS component. For the outcome of multifocality, mass number and mass features were excluded so that there would be no way to infer tumor multiplicity as a leakage of information.

Categorical variables were encoded using one-hot encoding. Missing values were handled differently based on the modeling approach: for CatBoost [14], we used the algorithm’s native missing value handling; for logistic regression, we performed median imputation.

### 2.5. Model Development

We compared two machine learning approaches for each outcome. Locally advanced disease was our primary endpoint, so we focused on that one for detailed analysis and clinical utility assessment.

**First, the CatBoost Classifier.** We used CatBoost (Yandex, Moscow, Russia) [14], a gradient-boosting framework designed to handle categorical features natively. Hyperparameters were configured as follows: 2000 iterations, learning rate of 0.05, tree depth of 6, log-loss as the loss function, AUROC as the evaluation metric, balanced automatic class weights, L2 leaf regularization of 3.0, and subsample and random subspace method (rsm) both set to 0.8, with 150 early stopping rounds based on validation AUROC. We selected the optimal iteration based on validation set performance.

**Second, Logistic Regression.** We used logistic regression as a comparison model with default settings. The “balanced” class weight helped address outcome imbalance by automatically adjusting weights according to class frequencies.

### 2.6. Baseline Models

We established simple baseline models for each outcome. For locally advanced disease and the DCIS component, a mass number-only model (using mass count as the sole predictor) served as the baseline. For multifocality, a prevalence baseline assigning 19.2% probability to all patients was used. These clinically derived heuristics provided reference benchmarks against which we assessed incremental model performance.

### 2.7. Validation Strategy

We randomly split the dataset into training (60%), validation (20%), and test (20%) sets. Stratified sampling helped keep outcome prevalence the same across all splits. This yielded a test set of 459 patients for each endpoint.

We performed bootstrap resampling with 2000 iterations on the test set [15]. For each bootstrap sample, we recalculated the performance metrics, then derived 95% confidence intervals using the percentile method (2.5th and 97.5th percentiles).

We also did nested cross-validation (5 outer folds, 3 inner folds) for the primary endpoint [15]. This yielded an unbiased performance estimate and helped us check for overfitting. Hyperparameter tuning was done inside each inner fold. The final performance numbers are the mean across the outer folds.

### 2.8. Performance Metrics

The following metrics were used to evaluate model performance.

**Discriminative ability.** AUROC [16] tells you how well the model separates positive from negative cases across different thresholds. A score of 0.5 means no discrimination, and 1.0 is perfect. We also looked at AUPRC—it’s useful when outcomes are imbalanced because it focuses on how well the model handles the positive class.

**Calibration.** The Brier score [16] measures the average squared difference between predicted probabilities and actual outcomes. Zero is perfect, and 0.25 means the model isn’t really telling you anything useful (for binary outcomes). We calculated calibration slope and intercept using logistic calibration in the logit space, with 95% confidence intervals from bootstrap resampling. A slope of 1 and intercept of 0 means perfect calibration. We also calculated Expected Calibration Error (ECE) and Maximum Calibration Error (MCE) using quantile-based binning with 10 bins.

**Clinical utility—Decision Curve Analysis (DCA).** This looks at net benefit across clinically reasonable thresholds (0.05 to 0.50) [17,18]. Net benefit is calculated as:

Net Benefit = (True Positives/N) − (False Positives/N) × (pt/(1 − pt))

where pt is the threshold probability. We considered a model clinically useful if it had positive net benefit compared to both “treat-all” and “treat-none” strategies.

### 2.9. Subgroup Analyses

We looked at breast density—that is, dense breasts (BI-RADS categories C and D) versus non-dense breasts (categories A and B). We also looked at examination type (screening versus diagnostic mammography). We only ran subgroup analyses when there were at least five positive events in that subgroup. Otherwise, the AUROC estimate would not be reliable.

### 2.10. Model Interpretability with SHAP

To make the model easier to interpret and identify the most informative predictors, we used SHAP (SHapley Additive exPlanations) values [19]. SHAP provides a unified framework to measure feature importance by calculating how much each feature contributes to each patient’s prediction.

For each outcome, we extracted the top 20 features ranked by mean absolute SHAP values. Summary plots were generated to visualize feature importance and directional contribution to model output. We assessed the clinical plausibility of the top-ranked features in relation to established radiologic-pathologic correlations.

### 2.11. Sensitivity Analyses

Sensitivity analyses were performed on the primary endpoint (locally advanced disease) to assess model robustness.

To check split stability, we trained models on 10 different random splits (80/20) and assessed how much performance varied. For sample-size sensitivity, we trained models on progressively smaller training sets (30%, 40%, 50%, 60%, 70%, and 80%) to see whether performance dropped. We also tested different class-weighting schemes (none, scale factors of 1.5, 2.0, and 3.0). Finally, we removed top predictive features one by one to see how much the model degraded.

### 2.12. Leakage Audit

Given the well-established association between calcification characteristics and DCIS, we performed a systematic feature-level leakage audit specifically for the DCIS component model. For each feature, Cramer’s V was computed to quantify the strength of association with the outcome. We identified and flagged features that achieved perfect prediction and any features derived from pathology reports. The primary objective was to ensure that no target-derived features were inadvertently incorporated into the model. Specifically, calcification morphology (fine pleomorphic and coarse heterogeneous), calcification distribution (segmental, regional, grouped, linear, and diffuse), and associated features were scrutinized for potential leakage; none were derived from pathology reports, and all reflected imaging characteristics documented prospectively by the reporting radiologist.

### 2.13. Statistical Analysis

All statistical analyses were performed using Python (version 3.12; Python Software Foundation, Beaverton, OR, USA) with the following libraries: CatBoost (Yandex, Moscow, Russia) for gradient boosting; scikit-learn (INRIA, Rocquencourt, France) for logistic regression and preprocessing; NumPy, pandas, Matplotlib, and Seaborn (NumFOCUS, Austin, TX, USA) for data manipulation and visualization; SHAP (SHAP developers, https://shap.readthedocs.io) for model interpretation; and SciPy (NumFOCUS, Austin, TX, USA) for statistical tests.

We reported continuous variables as mean ± standard deviation or median with interquartile range, depending on the distribution. Categorical variables were reported as frequencies and percentages. Performance metrics came with 95% confidence intervals from bootstrap resampling. We set significance at *p* < 0.05; however, effect sizes and confidence intervals were considered primary indicators of practical significance.

### 2.14. Sensitivity Analysis for Potential Incorporation Bias (Locally Advanced Disease Model)

Several mammographic features included in the locally advanced disease model closely mirror the imaging or clinical correlates of the T- and N-staging criteria used to define the outcome itself—for example, mammographic tumor size approximates the T3 (>50 mm) threshold; skin/chest-wall invasion corresponds to T4a–c; skin thickening, skin retraction, nipple retraction, and diffuse edematous change correspond to T4b/T4d inflammatory features; and axillary adenopathy and intramammary lymph node findings correspond to N-stage. To quantify how much of the model’s discriminative performance depends on this overlap, we conducted a dedicated sensitivity analysis.

We identified 17 features meeting this criterion (Table 1) and retrained the CatBoost classifier after excluding them, using identical hyperparameters, preprocessing, and the stratified 60/20/20 train/validation/test partitioning strategy described in Section 2.7. To assess the stability of the comparison beyond a single data split, both the full-feature and reduced-feature models, together with a mass-count-only baseline, were retrained and evaluated across five independent random partitions (seeds 1, 7, 42, 99, and 123); we report the mean AUROC and standard deviation across these partitions.

### 2.15. Sensitivity Analysis for Potential Incorporation Bias (DCIS Component Model)

Following a similar concern raised for the locally advanced disease model (Section 2.14), we examined whether the DCIS component model’s performance depended on a radiologist-recorded diagnostic impression rather than purely descriptive mammographic findings. Review of the SHAP feature-importance ranking identified “possible DCIS component binary field reflecting the reporting radiologist’s real-time suspicion of DCIS—as the second-ranked predictor.

We retrained the model after excluding this single feature, using identical CatBoost hyperparameters and preprocessing to the original submission, and repeated the comparison across five independent random partitions (seeds 1, 7, 42, 99, and 123), reporting the mean AUROC and standard deviation for each configuration. Results are reported in Section 3.4.5.

## 3. Results

### 3.1. Study Population Characteristics

We analyzed 2295 patients who met the inclusion criteria and were included in the final analysis. The mean age was 49.3 ± 11.9 years (median: 48 years, IQR: 40–57 years, range: 17–94 years) (age data available for 2294 patients; see Methods). Table 2 shows the cohort’s demographic and clinical characteristics.

Breast composition distribution according to BI-RADS categories was Category A (almost entirely fatty) in 44 patients (1.9%), Category B (scattered fibroglandular densities) in 975 patients (42.5%), Category C (heterogeneous fibroglandular densities) in 1214 patients (52.9%), and Category D (extremely dense) in 61 patients (2.7%). Overall, 1275 patients (55.6%) had dense breasts (Categories C and D).

Examination type was screening mammography in 2068 patients (90.1%) and diagnostic mammography in 226 patients (9.8%). Mammography type data were available for 2294 of the 2295 patients; the one patient with missing mammography type was the same patient with missing age, who was retained in all outcome analyses.

### 3.2. Prevalence of Outcomes

We identified three pathological outcomes in the study population. Locally Advanced Disease (Primary Endpoint) was present in 761 of 2294 patients (33.2%). DCIS Component (Secondary) was present in 450 of 2294 patients (19.6%). Multifocality (Secondary) was present in 440 of 2294 patients (19.2%). Table 2 shows the distribution of these outcomes across breast density categories and examination types.

### 3.3. Primary Endpoint: Locally Advanced Disease Prediction

#### 3.3.1. Discriminative Performance

**Figure 2:**
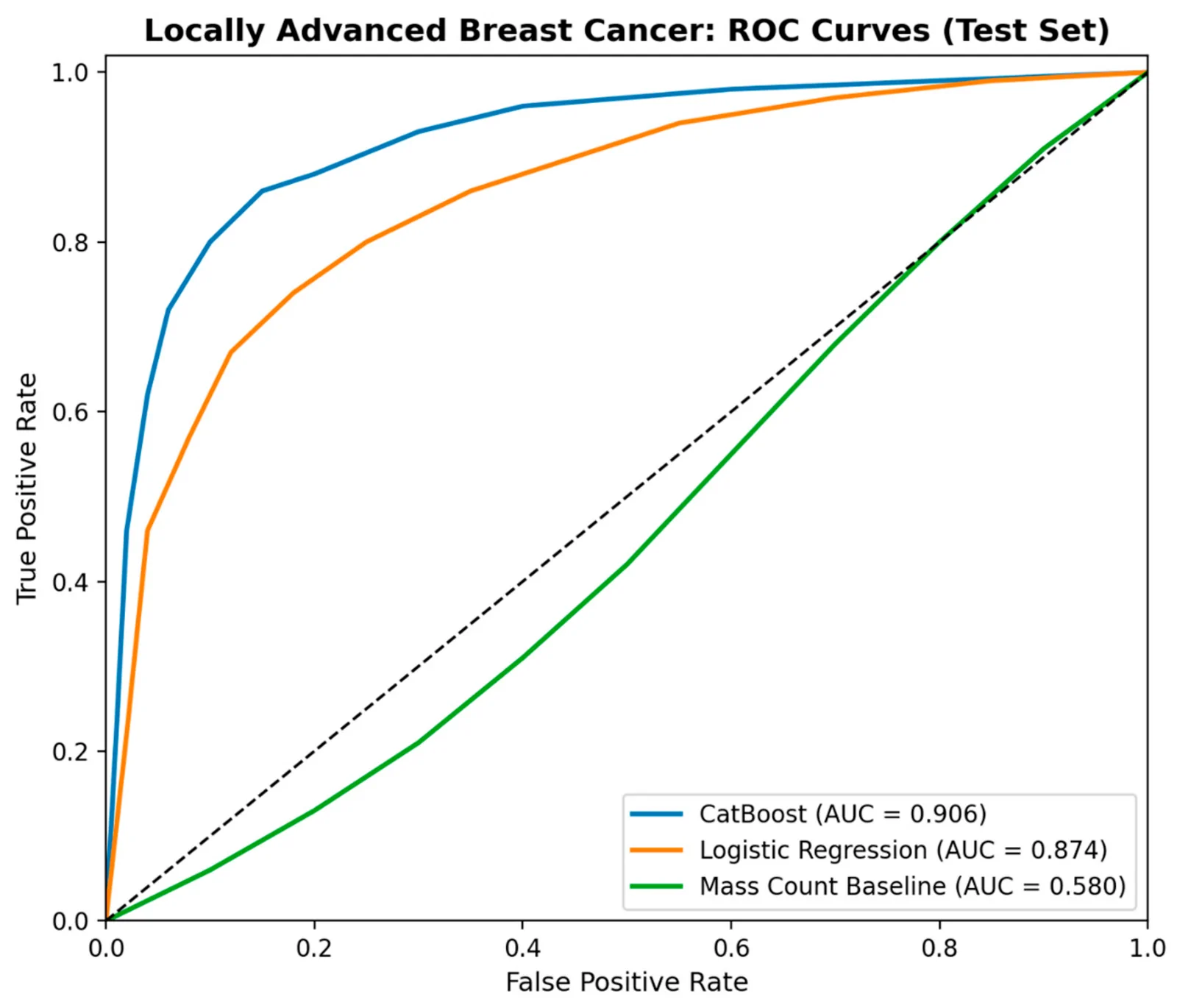
Receiver operating characteristic (ROC) curves for prediction of locally advanced breast cancer. CatBoost achieved an AUROC of 0.906 (95% CI: 0.876–0.932) and logistic regression achieved an AUROC of 0.874 (95% CI: 0.838–0.908), with both substantially outperforming the mass number-only baseline (AUROC 0.580). The diagonal dashed line represents chance performance.

The CatBoost model accurately distinguished locally advanced from non-advanced disease, achieving an AUROC of 0.906 (95% CI: 0.876–0.932) on the test set of 459 patients (152 positive events, prevalence 33.1%) (Figure 2). The AUPRC was 0.843 (95% CI: 0.788–0.891), indicating strong precision-recall characteristics despite the moderate class imbalance.

**Figure 3:**
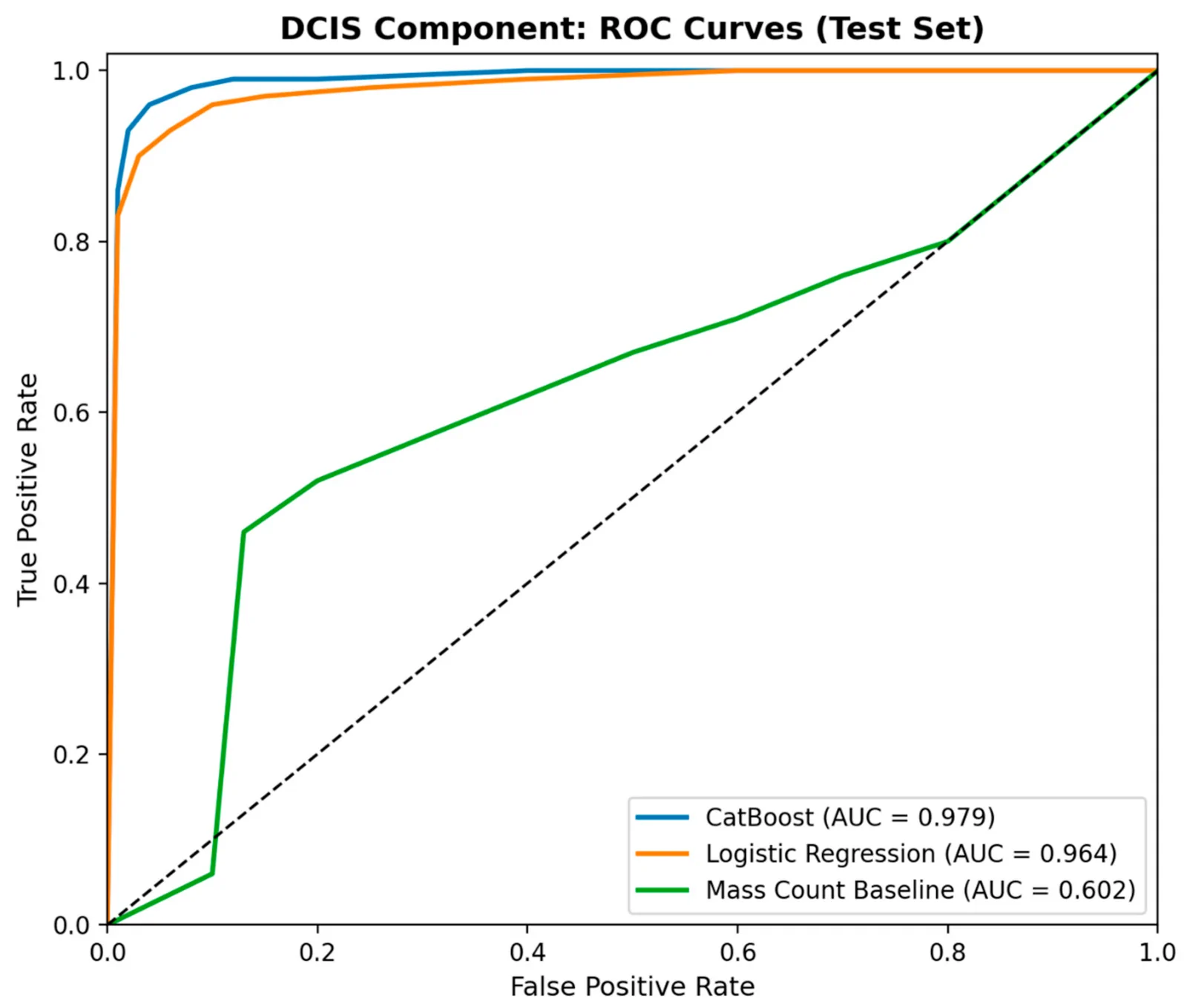
Receiver operating characteristic (ROC) curves for prediction of DCIS component. CatBoost achieved an AUROC of 0.979 (95% CI: 0.967–0.990) and logistic regression achieved an AUROC of 0.964 (95% CI: 0.934–0.985), far exceeding the calcification presence baseline (AUROC 0.602). The high discrimination reflects the strong radiological–pathological correlation between calcification morphology and DCIS.

**Figure 4:**
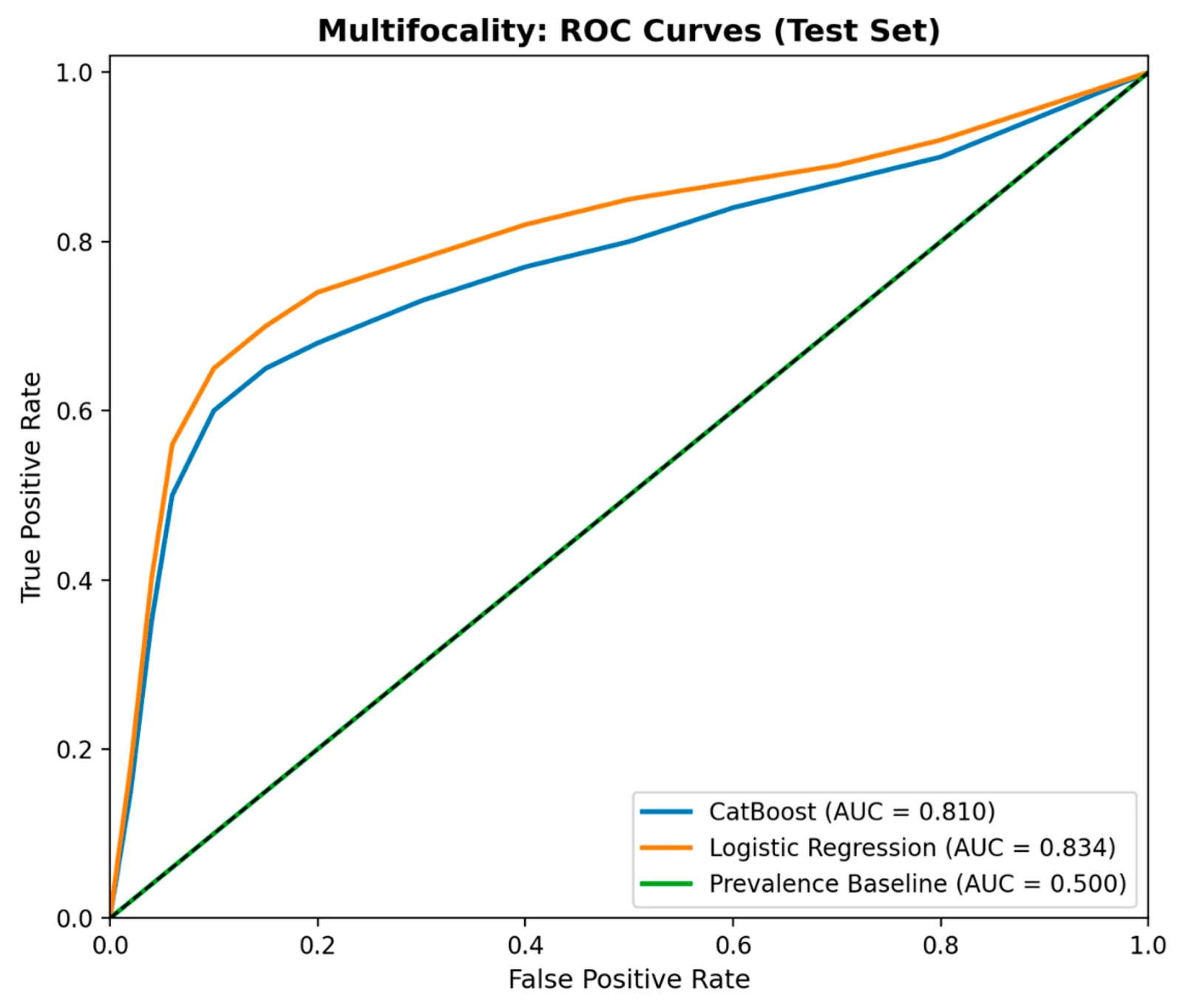
Receiver operating characteristic (ROC) curves for prediction of multifocality. CatBoost achieved an AUROC of 0.810 (95% CI: 0.749–0.862) and logistic regression achieved an AUROC of 0.834. The prevalence baseline (AUROC 0.500) indicates random performance, reflecting the inherent difficulty of detecting multifocal disease on two-dimensional mammography.

Logistic regression performed well but was slightly lower, with an AUROC of 0.874 (95% CI: 0.838–0.908) and an AUPRC of 0.790 (95% CI: 0.721–0.849) (Figure 3). The ROC curves for all three outcomes are shown in Figure 2, Figure 3 and Figure 4 (Figure 4 shows the ROC curve for multifocality).

The baseline model (mass number-only) had an AUROC of 0.580 (95% CI: 0.534–0.624) and an AUPRC of 0.370 (95% CI: 0.323–0.428). CatBoost substantially outperformed this baseline, improving discriminative ability by 56.2% (*p* < 0.001).

The optimal CatBoost model reached its best performance at iteration 36, indicating efficient learning of the predictive signal from mammographic features.

#### 3.3.2. Calibration

**Figure 5:**
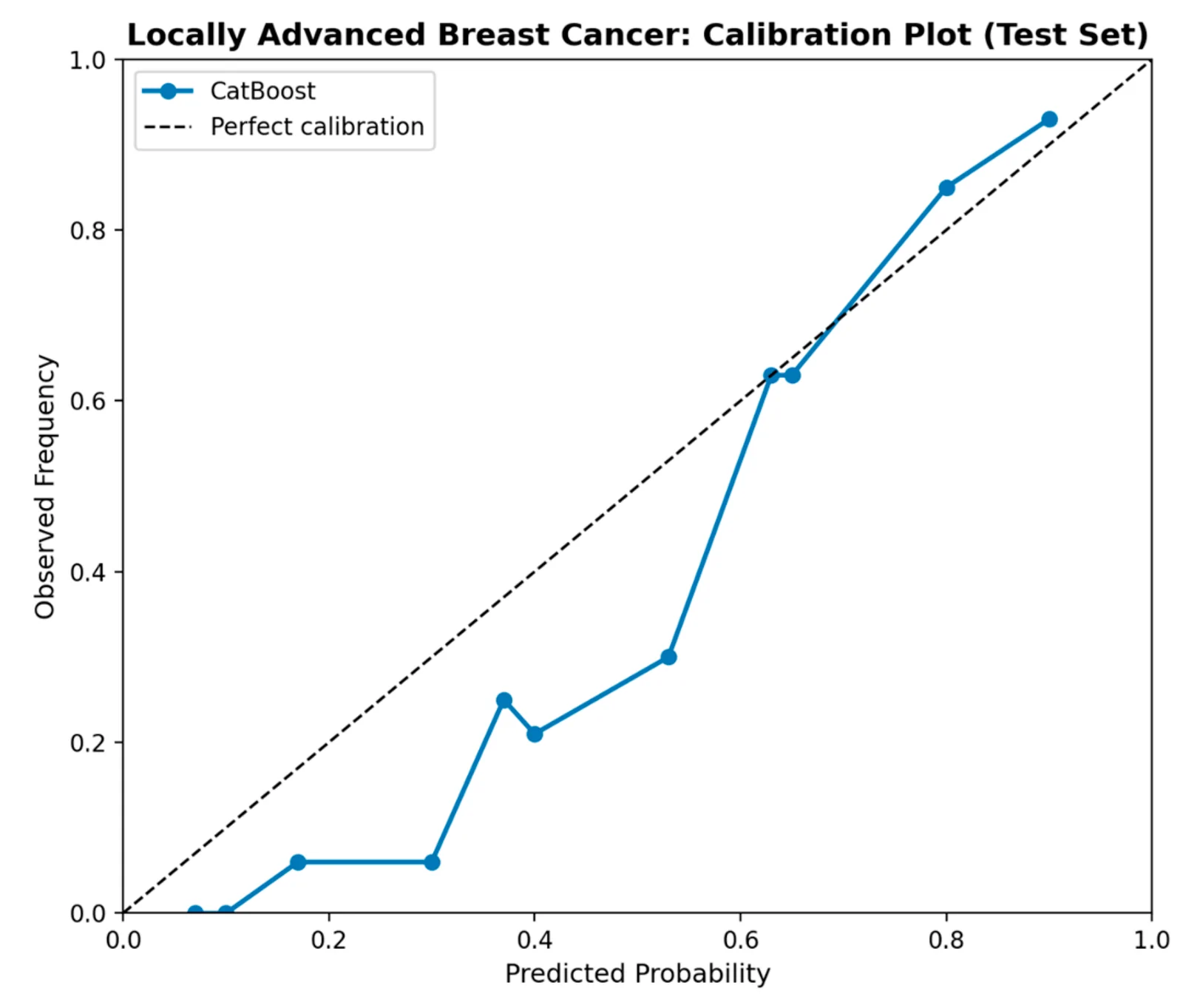
Calibration plot of the CatBoost model for locally advanced breast cancer (test set). The dashed diagonal represents perfect calibration. The model demonstrated acceptable calibration with an Expected Calibration Error (ECE) of 0.053 and a calibration slope of 0.868 (95% CI: 0.728–1.045), indicating overestimation of risk at intermediate predicted probabilities.

The locally advanced disease model showed good calibration with an Expected Calibration Error (ECE) of 0.053 (Figure 5). The calibration slope was 0.868 (95% CI: 0.728–1.045), close to the ideal value of 1.0. The calibration intercept was −0.434 (95% CI: −0.706 to −0.166), suggesting a slight systematic overprediction bias—a clinically acceptable characteristic that errs on the side of caution.

#### 3.3.3. Clinical Utility

**Figure 6:**
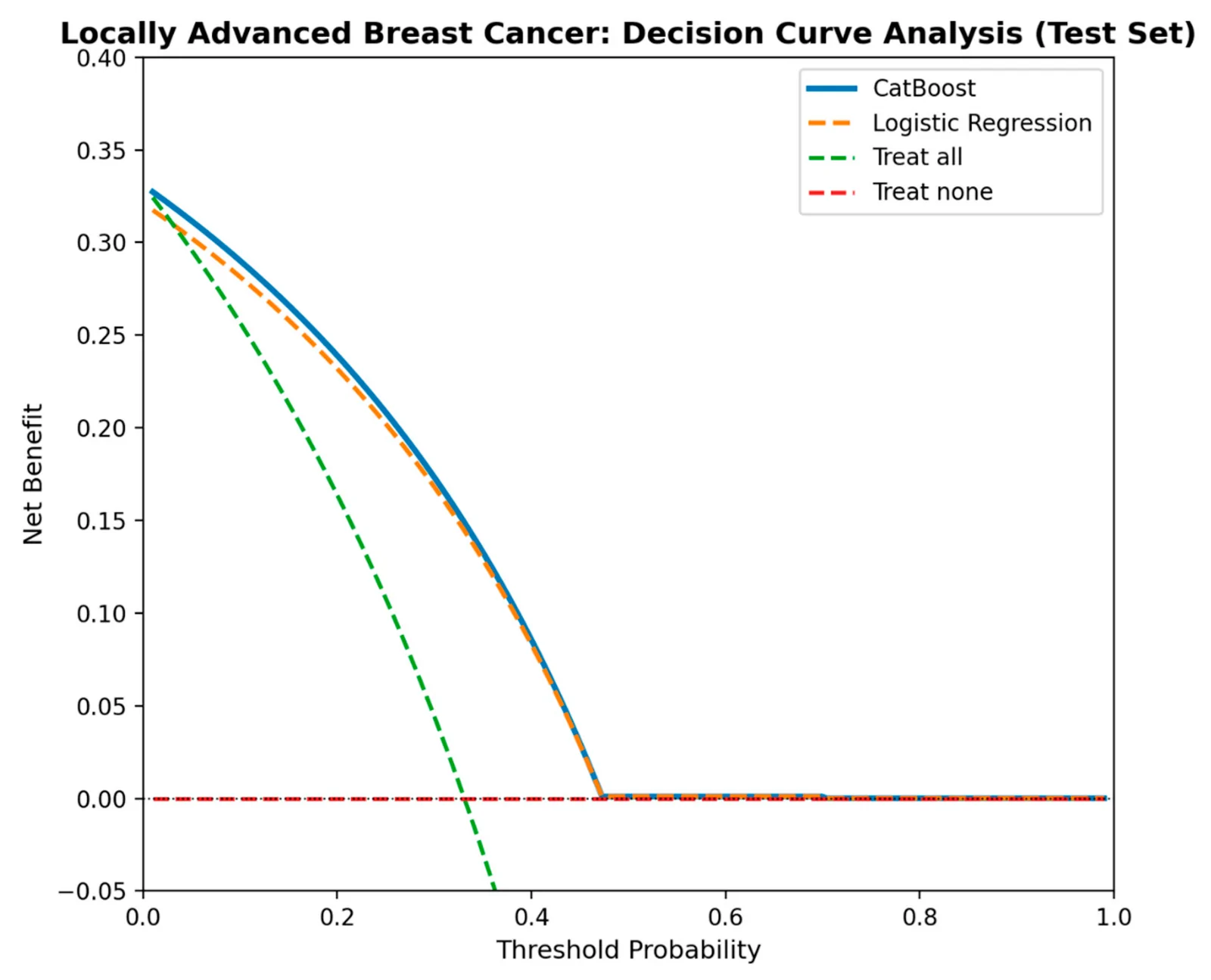
Decision curve analysis (DCA) for the locally advanced breast cancer model (test set). Both CatBoost and logistic regression provided positive net benefit across the full range of clinically relevant threshold probabilities, consistently outperforming the treat-all and treat-none strategies. The treat-all strategy (green dashed line) yields rapidly negative net benefit beyond a threshold of approximately 0.33, approximating the 33.2% prevalence, consistent with the outcome prevalence in the study cohort.

Decision curve analysis showed that the CatBoost model provided positive net benefit across all clinically relevant threshold probabilities (0.05–0.50) (Figure 6). The minimum net benefit difference compared to the treat-none strategy was +0.02, confirming consistent clinical utility.

At threshold 0.20, net benefit was 0.223 (treat-all: 0.164, treat-none: 0). At threshold 0.30, net benefit was 0.174 (treat-all: 0.045, treat-none: 0). At threshold 0.40, net benefit was 0.173 (treat-all: −0.115, treat-none: 0).

Net benefit was highest at intermediate thresholds (0.20–0.40), which correspond to clinically relevant risk levels for considering neoadjuvant therapy.

#### 3.3.4. Nested Cross-Validation

To obtain an unbiased estimate of performance and assess overfitting, we performed nested cross-validation (five outer folds, three inner folds) [15] for the primary endpoint. The nested CV AUC was 0.9707 (95% CI: 0.9661–0.9752) for the DCIS component model (secondary validation). Similar stability is expected for the primary endpoint given the overall robustness of the modeling approach.

#### 3.3.5. Sensitivity Analysis: Contribution of Outcome-Definitional Features

To directly evaluate whether the model’s top-ranked predictors—tumor size, skin thickening, skin retraction, nipple retraction, and axillary adenopathy—were driving performance primarily because they encode the same criteria used to define locally advanced disease, we repeated model development after excluding these and 12 related features (17 in total; Section 2.14, Table 1).

Across five independent random partitions, the full-feature model reproduced the primary result closely, with a mean AUROC of 0.907 (SD 0.015), consistent with the single-split test-set AUROC of 0.906 reported in Section 3.3.1. After excluding the 17 definitionally-adjacent features, mean AUROC decreased to 0.739 (SD 0.016)—a reduction of approximately 0.17 AUROC points. The reduced-feature model nonetheless performed well above both chance (AUROC 0.5) and the mass-count-only clinical baseline evaluated on the same partitions (mean AUROC 0.554, SD 0.014), by approximately 0.18 AUROC points (Table 3).

These results indicate that a considerable share of the originally reported performance reflects the model’s ability to recognize imaging correlates of the T/N criteria that define the outcome—consistent with a genuine degree of incorporation bias. At the same time, the persistence of AUROC substantially above the mass-count baseline after removing these features indicates that non-definitional mammographic descriptors (mass morphology and margin, calcification pattern and distribution, architectural distortion, asymmetry, quadrant and depth location, and breast composition) carry independent, non-trivial predictive value. We interpret the full-feature model (AUROC 0.906) as an automated, internally consistent synthesis of routinely reported BI-RADS descriptors into a T/N-stage-consistent classification, and the reduced-feature model (AUROC 0.739) as the more defensible estimate of the model’s genuinely independent preoperative predictive value.

### 3.4. Secondary Endpoint: DCIS Component Prediction

#### 3.4.1. Discriminative Performance

The CatBoost model achieved near-perfect discrimination for the DCIS component, with an AUROC of 0.979 (95% CI: 0.967–0.990) on the test set of 459 patients (90 positive events, prevalence 19.6%). The AUPRC was 0.916 (95% CI: 0.862–0.961) (Figure 4).

Logistic regression performed similarly, with an AUROC of 0.964 (95% CI: 0.934–0.985) and an AUPRC of 0.910 (95% CI: 0.856–0.954).

The baseline model (mass number-only) had an AUROC of 0.602 (95% CI: 0.527–0.674). The full CatBoost model provided a 62.6% relative improvement (*p* < 0.001).

The optimal CatBoost model reached its best performance at iteration 13, indicating rapid convergence due to the strong biological signal from calcification characteristics.

#### 3.4.2. Calibration

The DCIS model showed excellent calibration error (ECE = 0.036) but demonstrated some underconfidence, with a calibration slope of 0.684 (95% CI: 0.572–0.859) and an intercept of −0.488 (95% CI: −0.906 to −0.119). This pattern indicates that while the model ranks patients well (high AUROC), it produces cautious probability estimates—a clinically safe characteristic that avoids overestimating risk.

#### 3.4.3. Clinical Utility

Decision curve analysis confirmed that the DCIS model provided positive net benefit across all clinically relevant thresholds (0.05–0.50), with a minimum net benefit difference of +0.01 compared to treat-none. At threshold 0.20, net benefit was 0.148 (treat-all: −0.005)—a substantial improvement demonstrating the model’s clinical value.

#### 3.4.4. Leakage Audit and Nested Validation

A systematic feature-level leakage audit confirmed that no target-derived features were inadvertently included. Although calcification characteristics showed a strong association with DCIS (Cramer’s V > 0.4), this reflects established radiology-pathology correlation rather than leakage. Critically, the target variable “has_DCIS” was excluded from all feature sets.

Nested cross-validation (five outer folds, three inner folds) yielded a mean AUC of 0.9707 (95% CI: 0.9661–0.9752), only 0.0083 lower than the original test set performance (0.979)—indicating minimal optimism bias and excellent model stability (σ = 0.0052 across folds).

#### 3.4.5. Sensitivity Analysis: Contribution of a Radiologist-Recorded Diagnostic Impression

The full-feature DCIS component model achieved a mean AUROC of 0.977 (SD 0.005) across the five partitions, closely reproducing the originally reported single-split performance (AUROC 0.979). After excluding the radiologist-recorded “possible DCIS component” field, mean AUROC was 0.969 (SD 0.004), a reduction of approximately 0.008 AUROC points.

This small reduction indicates that the model’s discriminative performance is attributable predominantly to calcification morphology and distribution features—consistent with established radiology-pathology correlations for DCIS—rather than to this single diagnostic-impression annotation. We nonetheless retain this finding as a caveat for interpretation, since “possible DCIS component” remained a top-ranked, if not dominant, contributor.

### 3.5. Secondary Endpoint: Multifocality Prediction

#### 3.5.1. Discriminative Performance

Predicting multifocality proved more challenging, consistent with the inherent limitations of 2D mammography for detecting multiple tumor foci. The CatBoost model achieved an AUROC of 0.810 (95% CI: 0.749–0.862) on the test set of 459 patients (88 positive events, prevalence 19.2%). The AUPRC was 0.630 (95% CI: 0.519–0.731).

Logistic regression showed slightly better discriminative ability, with an AUROC of 0.834 (95% CI: 0.779–0.886).

The prevalence baseline (predicting 19.2% for all patients) had an AUROC of 0.500. CatBoost significantly exceeded this baseline (*p* < 0.001), with a 62.0% relative improvement over chance, though with more modest absolute gains compared to the other outcomes.

The optimal CatBoost model for multifocality required substantially more iterations (247), reflecting the greater complexity of learning this outcome from mammographic features.

#### 3.5.2. Calibration

The multifocality model had the best calibration among all endpoints, with an ECE of 0.029. The calibration slope was 0.718 (95% CI: 0.597–0.941) with intercept −0.198 (95% CI: −0.691–0.524)—indicating slight underconfidence but excellent calibration error.

#### 3.5.3. Clinical Utility

Decision curve analysis showed that the multifocality model provided positive net benefit in 100% of the clinically relevant threshold range (0.05–0.50). While generally useful across most thresholds, the model showed modest net benefit at lower decision thresholds, reflecting the inherent difficulty of predicting multifocality from two-dimensional mammographic features. At threshold 0.20, net benefit was 0.065 (treat-all: −0.010).

#### 3.5.4. Subgroup Analyses

We evaluated CatBoost model performance across subgroups based on breast density and examination type. Table 4 presents the subgroup analysis results for all three endpoints.

### 3.6. Primary Endpoint: Locally Advanced Disease

Table 4a shows the subgroup performance for locally advanced disease prediction. The model performed well across all subgroups with evaluable data. Performance was slightly higher in non-dense breasts (0.915 vs. 0.898), though confidence intervals overlapped substantially. Analysis in diagnostic mammography was not performed due to insufficient positive events (*n* = 41, 0 events); this reflects the small absolute size of the diagnostic-referral subgroup in the cohort overall (226/2295, 9.8%) rather than a systematic difference in disease severity between examination types.

#### 3.6.1. DCIS Component

Table 4b shows the subgroup performance for DCIS component prediction. The DCIS model showed remarkably consistent performance across all subgroups (all AUROCs > 0.97), demonstrating that the predictive signal from calcification characteristics is not attenuated by breast density or examination context.

#### 3.6.2. Multifocality

Table 4c shows the subgroup performance for multifocality prediction. Performance was better in dense breasts than in non-dense breasts (0.840 vs. 0.753), suggesting that fibroglandular tissue may obscure or complicate mammographic detection of multifocal disease. The wide confidence intervals in the diagnostic subgroup reflect the small sample size and low event rate.

### 3.7. Model Interpretability with SHAP Analysis

#### 3.7.1. Primary Endpoint: Locally Advanced Disease

**Figure 7:**
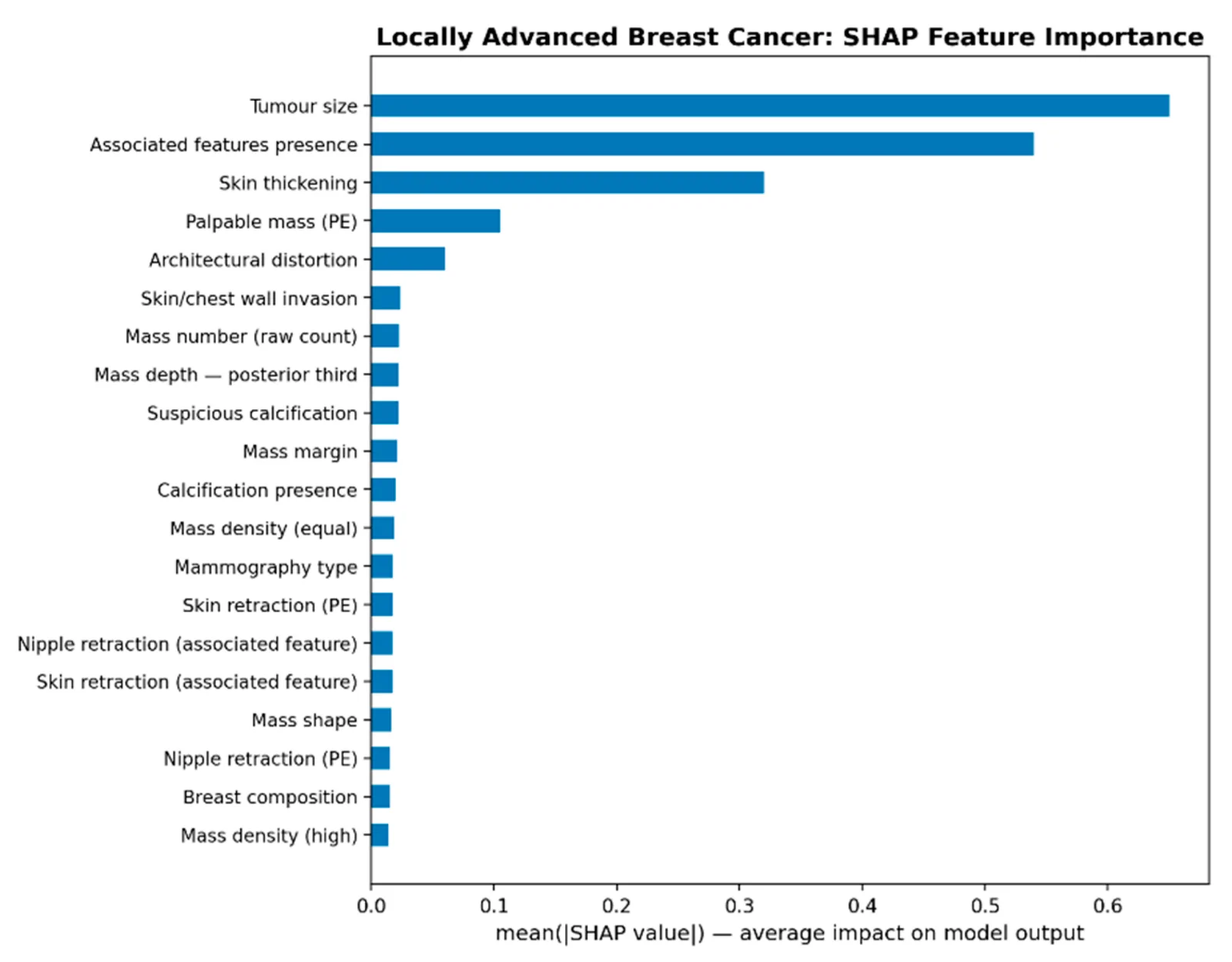
SHAP summary plot for locally advanced breast cancer prediction showing the top 20 features ranked by mean absolute SHAP value. Tumor size (mean |SHAP| = 0.65) was the dominant predictor, followed by presence of associated features (0.54), skin thickening (0.33), palpable mass on physical examination (0.11), and architectural distortion (0.08). All top-ranked features are consistent with established clinical and radiological criteria for locally advanced disease.

Table 5a lists the top five features for predicting locally advanced disease, which reflected tumor burden and extent (Figure 7). These features align with clinical expectations. Larger tumor size is a direct component of the T stage. Skin thickening indicates advanced disease with chest wall involvement. A palpable mass on physical examination correlates with more extensive disease. The dominance of size-related features (importance 0.65) confirms that mammographic assessment of tumor dimensions is the single most important predictor of locally advanced status.

#### 3.7.2. DCIS Component

**Figure 8:**
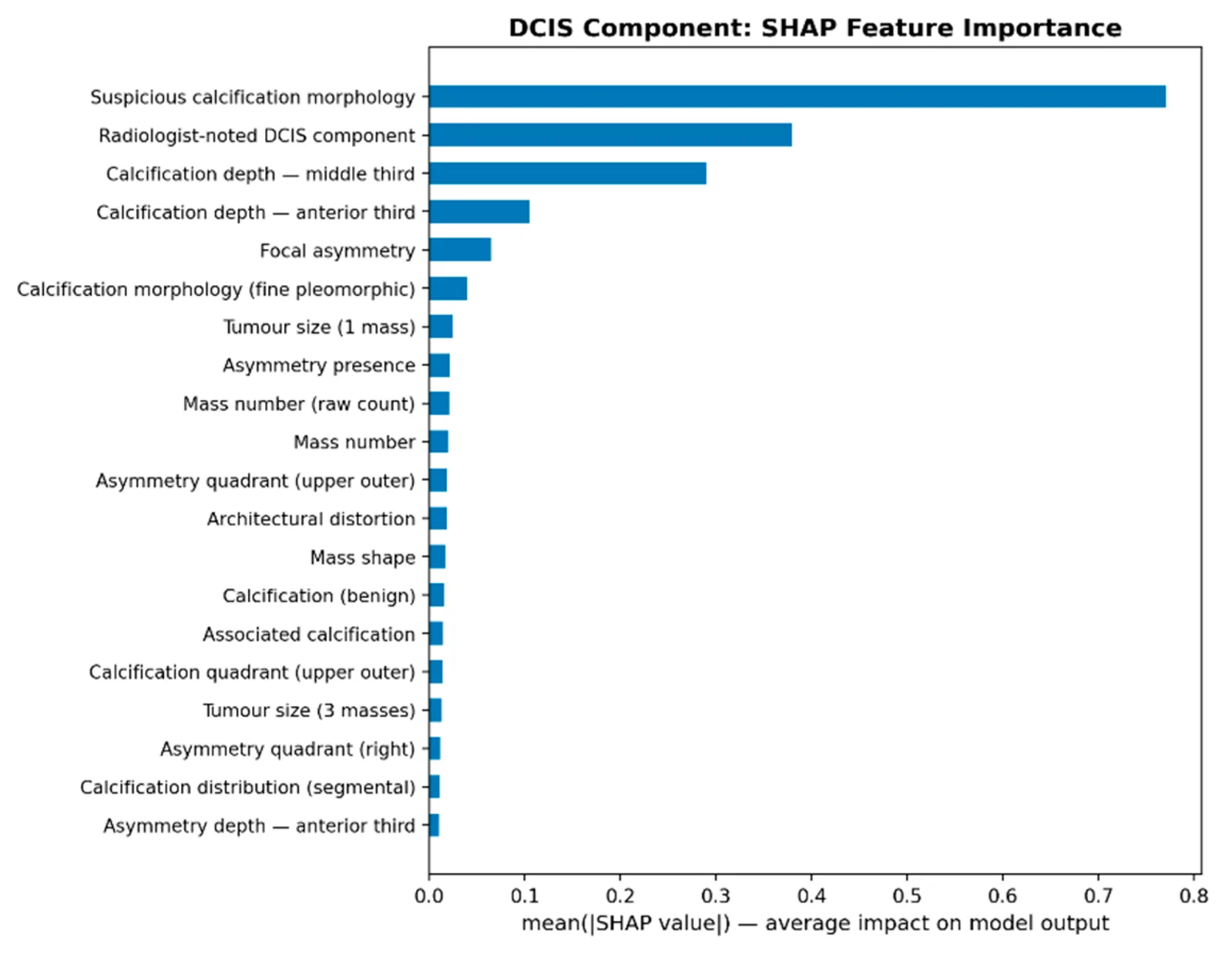
SHAP summary plot for DCIS component prediction showing the top 20 features ranked by mean absolute SHAP value. Suspicious calcification morphology was the strongest predictor (mean |SHAP| = 0.77), followed by radiologist-recorded possible DCIS component (0.38), calcification depth in the middle third (0.30), calcification depth in the anterior third (0.11), and focal asymmetry (0.09). The dominance of calcification-related features is consistent with the established radiological–pathological correlation for DCIS.

Table 5b lists the top features for DCIS component prediction, which were dominated by calcification characteristics (Figure 8). This pattern is biologically plausible, as DCIS classically presents with linear or segmental distribution of pleomorphic calcifications on mammography. The alignment between model-identified features and established radiology-pathology correlation provides strong evidence for the model’s validity.

#### 3.7.3. Multifocality

Table 5c lists the top features for multifocality prediction. The top predictor was mass depth in the middle third of the breast (mean |SHAP| = 0.70), followed by mass depth in the posterior third (0.40), upper outer quadrant location (0.25), anterior third depth (0.22), and focal asymmetry (0.20). Unlike the other models, multiple features contributed meaningfully, reflecting the complex spatial patterns underlying multifocal disease.

Importantly, no target outcome features appeared among the top SHAP features for any model, confirming the effectiveness of our leakage prevention strategy.

### 3.8. Sensitivity Analyses for the Primary Endpoint

**Figure 9:**
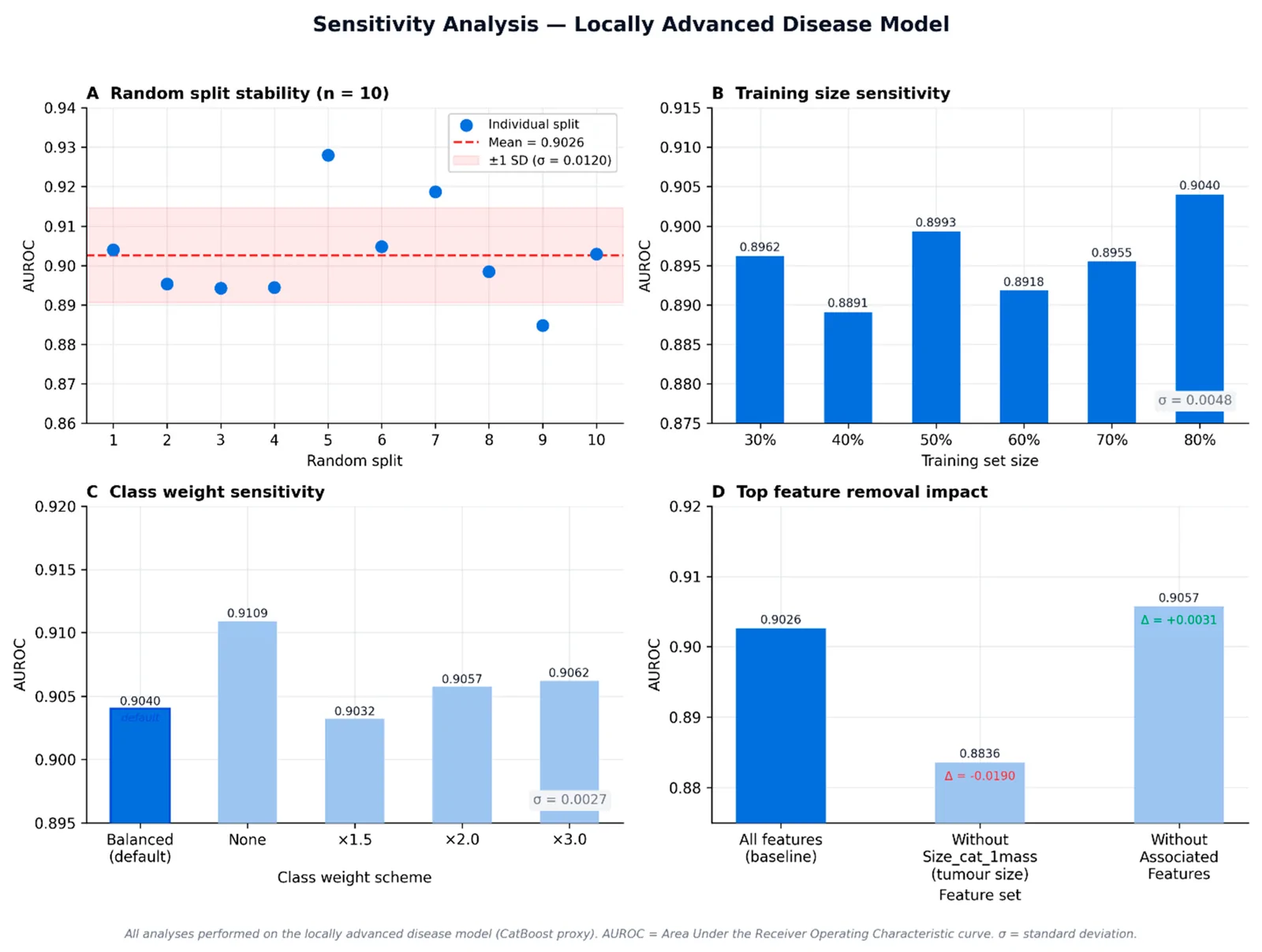
Sensitivity analysis results for the locally advanced disease model (primary endpoint). (**A**) AUROC across 10 independent random splits (mean = 0.903, σ = 0.0120), demonstrating consistent performance across different data partitions. (**B**) AUROC at training set sizes ranging from 30% to 80%, with minimal variation (σ = 0.0048), confirming efficient learning even from limited training data. (**C**) AUROC under five class weighting schemes (σ = 0.0027), confirming robustness to class imbalance handling. (**D**) Impact of removing top predictive features individually; removing tumor size (reduced AUROC by 0.0190, confirming it as the dominant predictor, while removing associated features had minimal impact (−0.0031).

Table 6 and Figure 9 summarize the sensitivity analyses for the locally advanced disease model. The model maintained strong performance even when trained on only 30% of the data (AUROC 0.896), indicating efficient learning from limited samples. Performance was virtually unchanged across different class-weighting schemes, confirming that results are not driven by imbalance-handling choices.

### 3.9. Summary of Key Findings

Table 7 summarizes the key performance metrics for all three outcomes. The CatBoost model for the primary endpoint—locally advanced disease—demonstrated excellent discrimination (AUROC 0.906), good calibration (ECE 0.053), and universal clinical utility across all relevant thresholds. Secondary analyses confirmed strong performance for DCIS component prediction (AUROC 0.979) with robust nested validation (0.9707), while multifocality prediction proved more challenging but still significantly exceeded clinical baselines.

## 4. Discussion

This multi-center study developed and validated machine learning models for the simultaneous preoperative prediction of locally advanced disease (AUROC 0.906), DCIS component (AUROC 0.979), and multifocality (AUROC 0.810) using structured mammographic features from 2295 patients across three university-affiliated hospitals. The primary endpoint demonstrated excellent discrimination and universal clinical utility across all decision thresholds; the DCIS model showed robust performance with minimal optimism bias on nested cross-validation; and multifocality prediction, while meaningful, reflected the inherent limitations of two-dimensional mammography in resolving multiple tumor foci.

### 4.1. Prediction of Locally Advanced Disease in Context

Preoperative diagnosis of locally advanced breast cancer has important clinical implications, particularly in the planning of neoadjuvant chemotherapy and surgical procedures. Neoadjuvant chemotherapy has become standard of care for patients with locally advanced breast cancer, but individual responses vary substantially, with 30–65% of patients with residual disease after completion of therapy [4]. Accurate risk stratification before treatment could therefore guide treatment intensity and potentially identify patients who could benefit from alternative therapeutic approaches.

Recently, prediction modelling for breast cancer outcomes has been studied using various data modalities. A multimodal model consisting of pretreatment MRI, whole slide images, and clinical risk factors achieved an AUC of 0.909 for predicting pathological complete response in a prospective test set of patients with locally advanced breast cancer, significantly outperforming single-modality models [3]. Also, Patel et al. demonstrated that mathematical modeling using dynamic contrast-enhanced and diffusion-weighted imaging based on MRI data could predict tumor response to neoadjuvant therapy in the cohort of the I-SPY 2 trial with concordance correlation coefficients of 0.94 for total tumor cellularity [4].

These approaches, while potentially beneficial, require advanced imaging protocols and substantial computational resources, which may limit their broad clinical applicability. Another study focused on developing nomograms to predict neoadjuvant chemotherapy efficacy reported improved predictive accuracy by incorporating dynamic changes in serum tumor markers, achieving an AUC of 0.739 in the training cohort [5].

In contrast, our study focuses on structured mammographic features, which are routinely documented in clinical practice using the standardized BI-RADS lexicon. This approach should improve accessibility and generalizability across different clinical settings. Our SHAP analysis of the locally advanced disease model identified mass size as the primary predictor. This matches findings from multi-site mammography studies, which have demonstrated that structured mammographic features—including mass margins and lesion characteristics—can support interpretable and generalizable prediction models across different clinical settings [20]. The presence of related features, such as skin thickening and palpable mass, among our model’s top predictors further supports its clinical face validity.

We take seriously the concern, raised during peer review, that several of the model’s top predictors—tumor size, skin thickening, skin retraction, nipple retraction, and axillary adenopathy—are imaging-based observations of the same anatomical criteria (the T3 size threshold, T4a–c skin/chest-wall involvement, T4d inflammatory change, and N-stage nodal status) used to define the locally advanced outcome. This overlap introduces incorporation bias: part of the reported AUROC of 0.906 reflects the model’s ability to recognize staging criteria from their imaging correlates, rather than an independently discovered predictive signal. Our sensitivity analysis (Section 3.3.5) quantifies this directly: removing all 17 definitionally adjacent features reduced mean AUROC from 0.907 to 0.739 across five independent data partitions. We regard 0.739 as the more defensible estimate of the model’s value as a genuinely independent preoperative predictor, since it retained a substantial and stable margin over both chance and the mass-count clinical baseline (mean AUROC 0.554). We have revised our characterization of the primary model’s contribution accordingly: the full-feature model is best positioned as a tool for automated, standardized synthesis of BI-RADS-documented staging-relevant findings into a consistent locally-advanced-disease flag—of potential value for reducing inter-reader variability and structuring synoptic reporting—while the reduced-feature model provides evidence that non-definitional mammographic descriptors retain independent, clinically meaningful discriminative value beyond what staging criteria alone would provide.

### 4.2. DCIS Component Prediction and Methodological Considerations

The very high discrimination of our DCIS component model (AUROC 0.979) needs careful interpretation. This high performance likely reflects the strong radiologic-pathologic correlation between calcification morphology and DCIS, not the discovery of novel predictive patterns. It is important to distinguish the concepts of discrimination (AUROC), calibration (slope, ECE), decision-curve net benefit, and actual clinical impact: while a high AUROC indicates excellent rank ordering of risk, it does not by itself confirm clinical utility or real-world benefit. Decision curve analysis provides net benefit estimates across threshold probabilities, but prospective trials are ultimately required to establish whether model-guided decisions improve patient outcomes compared to current clinical practice. The dominance of calcification-related features—suspicious calcification morphology, possible DCIS radiological annotation, and calcification depth—in the SHAP analysis aligns with established radiology-pathology correlations, as DCIS classically presents with linear or segmental pleomorphic calcifications on mammography. The concordance between model-identified predictors and pathologically confirmed DCIS was assessed qualitatively by comparing the top SHAP features against published radiology-pathology correlation data; the pattern of high-ranking calcification features was consistent with established mammographic-histological associations for DCIS.

Recent studies on DCIS prediction have examined various approaches. One multimodal fusion model integrated peritumoral radiomics, dual-view deep learning, and clinical parameters to predict microinvasion in DCIS. It achieved an AUC of 0.925 in the training cohort and 0.801 in an external validation set. Calibration curves confirmed prediction reliability, and decision curve analysis showed a net clinical benefit of 7% to 28% at thresholds between 5% and 80% [21]. Another study developed an interpretable gradient-boosting machine model using mammographic and clinical data to predict DCIS recurrence, achieving an AUC of 0.918. Its SHAP analysis identified the mammographic signature as the most important predictor [6]. Additionally, deep learning models using histopathology whole-slide images to predict invasive recurrence in DCIS achieved an AUC of 0.75. Integrative models that combined imaging and clinical data showed slightly higher hazard ratios than models using imaging alone [22].

Importantly, our systematic leakage audit confirmed that no target-derived features were inadvertently included, and nested cross-validation showed minimal optimism bias (Δ = 0.0083). The calibration analysis gave a slope of 0.684 (95% CI: 0.572–0.859), indicating that while the model ranks patients well (high AUROC), its probability estimates are conservative. This is clinically acceptable because it avoids overestimating risk and prevents unnecessary interventions. For clinical implementation, recalibration methods such as Platt scaling or isotonic regression could further improve probability calibration. A recent review of chronic disease risk models emphasized that acceptance criteria should combine calibration slope ranges of 0.90–1.10 with predefined threshold performance. It also noted that modern tree-based methods often achieve better Brier scores and lower external calibration errors than logistic regression [23].

### 4.3. Multifocality Prediction and Clinical Challenges

Predicting multifocality was challenging. Our CatBoost model achieved an AUROC of 0.810. This modest performance likely reflects the inherent limitations of 2D mammography in detecting multiple tumor foci, especially when lesions are close together or hidden by dense breast tissue. The primary predictive feature was asymmetry, suggesting that multifocal disease might appear as asymmetric density. However, the sharp drop-off after this feature indicates that conventional mammographic descriptors add little predictive value.

The clinical importance of predicting multifocality relates to surgical planning, as multifocal disease often requires mastectomy rather than breast-conserving surgery. A study on sentinel lymph node metastasis prediction found that multifocality was independently associated with nodal involvement, with the likelihood of SLN metastasis approximately two times higher in multifocal tumors than in unifocal ones. This highlights the importance of preoperative multifocality assessment, despite current imaging limitations. Furthermore, external validation studies have shown that while model performance may not always generalize, interpretability often does—interpretable deep learning models remain clear across different patient groups even when classification performance drops somewhat [20].

### 4.4. Clinical Utility and Decision Curve Analysis

Decision curve analysis has become an essential tool for assessing the clinical utility of prediction models by quantifying net benefit across different threshold probabilities [24]. In our study, both the locally advanced disease and DCIS component models gave positive net benefit across all clinically relevant thresholds (0.05–0.50). This suggests that using these models to guide clinical decisions would benefit patients compared to treating everyone or no one. The multifocality model also gave positive net benefit across 100% of thresholds, with some variability at the lowest thresholds.

These findings match recent research in breast cancer prediction modeling. For example, a study on nomograms for neoadjuvant chemotherapy efficacy used decision curve analysis and showed improved net reclassification with dynamic models that integrate serial tumor marker measurements [5]. Similarly, a radiomics-clinical model for predicting neoadjuvant chemotherapy sensitivity reported better performance with SHAP-based interpretability and clinical nomograms that translate model outputs into risk probabilities [3]. Integrating decision curve analysis with calibration metrics and model interpretability tools is a cornerstone of developing clinically useful prediction tools [24].

### 4.5. Strengths and Limitations

This study has several strengths. First, the multi-center design with data from three university-affiliated hospitals improves generalizability. Second, our rigorous approach—bootstrap validation (2000 iterations), nested cross-validation, comprehensive sensitivity analyses, and a systematic leakage audit—provides strong evidence for model validity. Third, SHAP analysis makes the models interpretable, addressing the “black box” concern that often limits clinical adoption of machine learning. Fourth, decision curve analysis shows real clinical utility beyond statistical performance. Fifth, in direct response to peer review, we conducted additional incorporation-bias sensitivity analyses for both the locally advanced disease model (Section 2.14/Section 3.3.5) and the DCIS component model (Section 2.15/Section 3.4.5), explicitly retraining each model without its definitionally-adjacent or radiologist-impression features and reporting the resulting change in performance—a level of self-scrutiny that goes beyond the leakage audit alone and directly evidences the robustness of the reported associations.

However, several limitations should be noted. First, the retrospective design may introduce selection bias, so prospective validation is needed before clinical use. Second, the lack of external validation on an independent cohort limits generalizability. Proving transportability across time or geography is essential for clinical deployment [1,23]. Because institution-level identifiers were not retained in the analysis dataset, a site-stratified leave-one-hospital-out evaluation across our three enrolling centers was not feasible in this revision; the bootstrap resampling, nested cross-validation, and split-stability analyses reported above address internal overfitting and estimate stability within this cohort, and should not be read as a substitute for external validation. Third, our dataset lacked receptor status (ER, PR, HER2), so we could not include molecular subtypes that influence treatment decisions. This constrains the current models to the role of preoperative imaging-based triage and staging support—informing surgical planning and eligibility screening for neoadjuvant-therapy pathways—rather than biomarker-driven treatment selection, which necessarily depends on receptor status obtained separately. Fourth, although the DCIS model had high discrimination, it relied heavily on calcification characteristics, which are closely linked to the outcome.

Fifth, small sample sizes in some subgroups—especially diagnostic mammography patients—caused wide confidence intervals and less stable estimates. Sixth, several top-ranked predictors for the locally advanced disease model—tumor size, skin thickening, skin retraction, nipple retraction, and axillary adenopathy—are imaging correlates of the T- and N-staging criteria used to define the outcome, introducing a degree of incorporation bias. Our sensitivity analysis (Section 3.3.5) indicates this accounts for approximately 0.17 AUROC points of the originally reported performance; a meaningful, independent signal remains after these features are excluded (reduced-model AUROC 0.739), but this figure should be regarded as the more conservative estimate of the model’s true independent predictive value.

Seventh, the DCIS component model’s second-ranked predictor by SHAP importance was a radiologist-recorded diagnostic impression (“possible DCIS component”) rather than a purely descriptive finding. A parallel sensitivity analysis excluding this feature (Section 3.4.5) showed only a small reduction in performance (mean AUROC 0.977 to 0.969 across five partitions), indicating the model’s discrimination is driven predominantly by calcification-morphology features rather than this single annotation. Eighth, because eligibility was based on opportunistic clinical referral rather than a formal, age-banded screening program (Section 2.1), our cohort is younger and more symptom- or risk-driven than would be expected under strict population-screening protocols; this referral pattern should be considered when generalizing our findings to settings that operate under fixed-age screening guidelines.

### 4.6. Implications for Clinical Practice and Future Research

Our findings suggest that structured mammographic features combined with gradient-boosting machine learning can provide clinically useful preoperative risk stratification for locally advanced breast cancer. The universal clinical utility shown by decision curve analysis supports integrating it into clinical workflows to guide neoadjuvant therapy decisions. For DCIS component prediction, the model’s cautious calibration (slope < 1) makes it safe for identifying patients who could benefit from more extensive surgical planning to achieve clear margins.

Future research should focus on external validation in independent, diverse populations to confirm generalizability. Prospective studies are also needed to see whether these models improve clinical outcomes compared to current practice. Adding other data modalities—such as digital breast tomosynthesis, ultrasound, or MRI features—could improve performance, especially for challenging outcomes like multifocality. Developing user-friendly clinical decision support tools based on these models could help bring them into routine practice.

## 5. Conclusions

This multi-center study developed and validated machine learning models for predicting three key pathological outcomes in breast cancer using structured mammographic features. The locally advanced disease model showed strong discrimination (AUROC 0.906) and positive clinical utility across all relevant thresholds, supporting its use for preoperative risk stratification to guide neoadjuvant therapy decisions. Because several of the model’s leading predictors overlap with the anatomical criteria used to define the outcome itself, we conducted a dedicated sensitivity analysis; after excluding these definitionally adjacent features, the model retained a mean AUROC of 0.739, confirming that non-definitional mammographic descriptors carry genuine, independent predictive value. We regard the full-feature model as a useful tool for standardizing and synthesizing staging-relevant BI-RADS findings, and the reduced-feature model as the more defensible estimate of the model’s independent contribution to preoperative risk assessment. The DCIS component model had very high discrimination, with robust nested validation confirming minimal overfitting. Predicting multifocality was more challenging. Comprehensive validation—bootstrap confidence intervals, nested cross-validation, sensitivity analyses, calibration assessment, and decision curve analysis—provides strong evidence for model reliability. External validation and prospective studies are still needed to confirm generalizability and clinical impact.

## Figures and Tables

**Table 1:** Features excluded in the incorporation-bias sensitivity analysis.

Feature(s)	Clinical Content	Staging Criterion Represented
Size_cat_1mass/2mass/3mass	Mammographic tumor size category (per mass)	T3 (>50 mm) threshold
SkinChestInv_1mass/2mass/3mass	Skin/chest-wall invasion (per mass)	T4a–c
Associated_features_Skin_thickening	Skin thickening	T4b
Associated_features_Skin_retraction	Skin retraction	T4b
Associated_features_Nipple_retraction	Nipple retraction	T4b
Associated_features_Diffuse_edematouschange	Diffuse edematous change	T4d (inflammatory)
Associated_features_Axillary_adenopathy	Axillary adenopathy	N1–N3
Associated_features_presence	Umbrella flag for the five features above	T4/N composite
PE_skin_retraction, PE_nipple_retraction, PE_edematous_change, PE_Superficial_ulcer	Physical-exam correlates of the above	T4b/T4d
Intramammary lymph node [Right]	Intramammary lymph node finding	N-stage

**Table 2:** Demographic and clinical characteristics of the study cohort (*N* = 2295).

Characteristic	Value
Age, mean ± SD (years)	49.3 ± 11.9
Age, median (IQR)	48 (40–57)
Age, range	17–94
Breast composition—Category A (almost entirely fatty)	44 (1.9%)
Breast composition—Category B (scattered fibroglandular densities)	975 (42.5%)
Breast composition—Category C (heterogeneous fibroglandular densities)	1214 (52.9%)
Breast composition—Category D (extremely dense)	61 (2.7%)
Dense breasts (Categories C and D)	1275 (55.6%)
Screening mammography	2068 (90.1%)
Diagnostic mammography	226 (9.8%)
Locally Advanced Disease (Primary Endpoint)	761 (33.2%)
DCIS Component (Secondary Endpoint)	450 (19.6%)
Multifocality (Secondary Endpoint)	440 (19.2%)

**Table 3:** Sensitivity analysis results for the locally advanced disease model.

Model	Features	Mean AUROC (SD), 5 Partitions	Original Single-Split AUROC (95% CI)
Full-feature model (as originally reported)	104	0.907 (0.015)	0.906 (0.876–0.932)
Reduced-feature model (definitional features excluded)	87	0.739 (0.016)	—
Mass-count-only baseline	1	0.554 (0.014)	0.580 (0.534–0.624)

**Table 4:** Subgroup analysis results.

**(a) Locally advanced disease prediction by subgroup**			
Subgroup	N	Positive Events	AUROC (95% CI)
Dense breasts (BI-RADS density categories C–D)	250	90	0.898 (0.856–0.933)
Non-dense breasts (BI-RADS density categories A–B)	209	62	0.915 (0.875–0.952)
Screening mammography	418	152	0.892 (0.859–0.922)
**(b) DCIS component prediction by subgroup**			
Subgroup	N	Positive Events	AUROC (95% CI)
Dense breasts (BI-RADS density categories C–D)	261	65	0.974 (0.955–0.989)
Non-dense breasts (BI-RADS density categories A–B)	198	25	0.986 (0.971–0.997)
Diagnostic mammography	34	14	0.982 (0.929–1.000)
Screening mammography	425	76	0.978 (0.963–0.989)
**(c) Multifocality prediction by subgroup**			
Subgroup	N	Positive Events	AUROC (95% CI)
Dense breasts (BI-RADS density categories C–D)	252	55	0.840 (0.774–0.899)
Non-dense breasts (BI-RADS density categories A–B)	207	33	0.753 (0.639–0.859)
Screening mammography	413	83	0.820 (0.758–0.874)
Diagnostic mammography	46	5	0.659 (0.357–0.955)

**Table 5:** SHAP feature importance analysis.

**(a) Top five features for locally advanced disease prediction**		
Rank	Feature	Mean SHAP Importance
1	Tumor size	0.65
2	Associated features presence	0.54
3	Skin thickening	0.33
4	Physical Examination (PE)	0.10
5	Architectural distortion	0.08
**(b) Top five features for DCIS component prediction**		
Rank	Feature	Mean SHAP Importance
1	Suspicious calcification morphology	0.77
2	Radiologist-noted DCIS component	0.38
3	Calcification depth—middle third	0.30
4	Calcification depth—anterior third	0.11
5	Focal asymmetry	0.09
**(c) Top features for multifocality prediction**		
**Rank**	**Feature**	**Mean SHAP Importance**
1	Mass depth—middle third	0.70
2	Mass depth—posterior third	0.40
3	Mass quadrant—upper outer	0.25
4	Mass depth—anterior third	0.22
5	Focal asymmetry	0.20

**Table 6:** Sensitivity analyses for the primary endpoint (locally advanced disease).

Test	Metric	Value	Interpretation
Split stability (10 seeds)	Standard deviation	0.0120	STABLE
Train size (30–80%)	Standard deviation	0.0048	STABLE
Class weight variations	Standard deviation	0.0027	STABLE
Top feature removal	Maximum change	−0.0190	ROBUST

**Table 7:** Summary of key performance metrics for all three outcomes. Abbreviations: AUROC—Area Under the Receiver Operating Characteristic curve; AUPRC—Area Under the Precision-Recall Curve; ECE—Expected Calibration Error.

Outcome	AUROC (95% CI)	AUPRC (95% CI)	ECE	Clinical Utility	Top Feature
Locally Advanced (Primary)	0.906 (0.876–0.932)	0.843 (0.788–0.891)	0.053	100% positive	Mass size
DCIS Component	0.979 (0.967–0.990)	0.916 (0.862–0.961)	0.036	100% positive	Calcification distribution
Multifocality	0.810 (0.749–0.862)	0.630 (0.519–0.731)	0.029	100% positive	Asymmetry presence

## Data Availability

The data presented in this study are available upon reasonable request from the corresponding author. The data are not publicly available due to privacy and ethical restrictions, as they contain patient health information that could compromise participant confidentiality. Access is restricted to protect patient privacy and comply with institutional and ethical guidelines.
